# 

**Published:** 1917-04

**Authors:** 


					﻿PlUBUSHED MONTHLY.	ATLANTA	SUBSCRIPTION $1.GO
JOURNAL-RECGI®
OF MEDlOOt;
(Atlanta Medical and Surgical Journal, Established 1855.	/ £□ J V
and Southern Medical Record, Established 1870.	$ OjIU YC3T
ol. LXIV	Atlanta, Ga., April, 1917	No. 1
				

